# Biofilm development by potentially pathogenic non-pigmented rapidly growing mycobacteria

**DOI:** 10.1186/1471-2180-8-184

**Published:** 2008-10-17

**Authors:** Jaime Esteban, Nieves Z Martín-de-Hijas, Teemu J Kinnari, Guillermo Ayala, Ricardo Fernández-Roblas, Ignacio Gadea

**Affiliations:** 1 Department of Clinical Microbiology, Fundación Jiménez Díaz-UTE, Madrid, Spain; 2 Department of Statistics, University of Valencia, Valencia, Spain

## Abstract

**Background:**

A study to evaluate the biofilm-development ability in three different media (Middlebrook 7H9, sterile tap water and PBS-5% glucose) was performed with 19 collection strains from 15 different species on non-pigmented rapidly growing mycobacteria (NPRGM). A microtiter plate assay was developed to evaluate the percentage of covered surface of the microtiter plate wells in different days from day 1 to day 69.

**Results:**

All strains were able to develop biofilm in all the tested media. Middlebrook 7H9 showed the fastest growth, followed by sterile tap water and PBS-5% glucose. A sigmoid growth curve was detected in all the strains both in Middlebrook 7H9 and in sterile tap water. A difference could be detected for *Mycobacterium abscessus *in tap water, where it showed faster growth than all the other strains.

**Conclusion:**

Biofilm development seems to be a property of all the species of NPRGM and it depends on the nutrients present in the medium. The microtiter plate assay described here is a useful tool to evaluate differences in biofilm development among the different species of rapidly growing mycobacteria.

## Background

Non-pigmented rapidly growing mycobacteria (NPRGM) are among the most commonly isolated species of nontuberculous mycobacteria in clinical laboratories. Although most of the members of this group have been described as the cause of human infections, *Mycobacterium abscessus*, *Mycobacterium fortuitum *and *Mycobacterium chelonae *are the most frequently isolated species in these syndromes [1,2]. Among the broad spectrum of these infections, nosocomial diseases are the most important because they may have devastating outcomes [1,3]. Many of these infections are related to implantable medical devices.

NPRGM are also environmental organisms that can be found in many habitats [4,5]. Several studies have shown that these organisms can be recovered from different water sources, including biofilms present in plumbing systems. Biofilms are considered important in device-related infections due in part to their increased resistance to antimicrobials [6,7]. However, despite their importance as human pathogens, there are only a few *in vitro *studies about NPRGM. In this study we report the results of a series of experiments aimed to evaluate the ability of different strains of NPRGM (including all the clinically relevant species) to develop biofilm under different nutrient conditions, and the relationship of biofilm development with the presence of sliding motility among these strains.

## Methods

### Strains

The following strains were used in the experiments: *Mycobacterium fortuitum *ATCC 6841T and ATCC 13756, *Mycobacterium chelonae *ATCC 19235 and ATCC 35752T, *Mycobacterium abscessus *DSM 44196T, *Mycobacterium peregrinum *ATCC 14467T, *Mycobacterium mucogenicum *DSM 44124, *Mycobacterium septicum *ATCC 700731T, *Mycobacterium immunogenum *ATCC 700505T, *Mycobacterium mageritense *ATCC 700351T, *Mycobacterium porcinum *ATCC 33776T, *Mycobacterium senegalense *NCTC 10956T, *Mycobacterium elephantis *DSM 44368T, *Mycobacterium smegmatis *ATCC 607, ATCC 19420T and ATCC 14468, *Mycobacterium goodii *ATCC 700504T, *Mycobacterium alvei *ATCC 51304T, and *Mycobacterium brumae *ATCC 51384T. All strains were maintained frozen at -20°C until the experiments were performed.

### Sliding motility test

One colony of each mycobacteria was put in the centre of a plate of motility medium, consisting in Middlebrook 7H9 with 0.3% agar without supplements. The inoculated media were incubated at 37°C in a 5% CO_2 _atmosphere during 2 weeks [8]. The diameter of the bacterial growth was measured at days 4, 8, 12 and 16 using a digital caliper.

### Biofilm development test

After thawing, mycobacteria were checked for purity, inoculated on Middlebrook 7H9 broth and incubated at 30°C during 5 days. These broth cultures were centrifuged at 3000 × g, washed with sterile phosphate buffered saline (PBS) and resuspended and calibrated to a 0.5 McFarland Standard with PBS. Ninety-six well sterile flat-bottom tissue-culture treated polystyrene microtiter plates (Costar, USA) were inoculated with 100 μl of the suspension. Plates were incubated at 37°C during 30 minutes. The inoculum was then removed with a sterile Pasteur pipette. The wells were washed with sterile PBS and 100 μl of the following media were added for all the strains: Middlebrook 7H9, PBS-5% glucose, PBS- 5% glucose-0.5% glycerol and filter sterilized tap water. Plates were placed on an orbital shaker (80 rpm) and incubated at ambient room temperature for 69 days. The media were replaced on days 1, 4, 7, 11, 14, 18, 21, 25, 28, 32, 35, 39, 41, 44, 47, 51, 54, 58, 61, 65 and 69. On days 1, 4, 7, 11, 21, 28, 35, 41, 47, 54, 61 and 69, one well was washed with sterile distilled water and stained with basic fuchsine during 30 minutes, washed and decoloured for 10 seconds with absolute ethanol. The stained well was then photographed (3–4 images/well) at low magnification (10×) using a Leitz DM IL inverted microscope (Leica, Germany) with an attached Nikon Coolpix 8400 digital camera (Nikon, Japan). Each strain was tested at least two times in different experiments.

To check the viability of mycobacteria, randomly selected wells were analyzed by inoculating the culture medium onto tryptic soy agar-5% blood agar plates, which were incubated at room temperature during 3 days. All the experiment was repeated when a contamination was detected.

### Data analysis

Digital photographs obtained from the stained wells were analysed with the Image J software (National Institute of Health, Bethesda, MD, USA) to evaluate the surface covered by the biofilm. The proportion of surface covered by biofilm at each time point was used to construct a growth curve of the biofilm. The experiment was stopped when growth covered 100% of the surface.

### Confocal laser scanning microscopy (CLSM)

To evaluate the biofilm development, we randomly selected 4 strains (*M. fortuitum *ATCC 6841T, *M. septicum *ATCC 700731T, *M. smegmatis *ATCC 19420 and *M. immunogenum *ATCC 700505T) to be analysed by using confocal laser scanning microscopy (CLSM) using the following protocol:

One ml of a 0.5 McFarland turbidity inoculum was inoculated onto 6 × 4 well-plates (Nunc, USA) with a polystyrene plastic disc (Nunc, USA) at the bottom of each well. Plates were then incubated at room temperature using the same protocol that microtiter plates with Middlebrook 7H9 as culture medium. On days 7, 14 and 21, the medium was removed, and the plastic discs were stained with the Live/dead^© ^BacLight^© ^viability stain (Invitrogen, Eugene, OR, USA) according to instructions provided by the manufacturer. Stained discs were then analysed with a Leica DM IRB confocal laser scanning microscope (Leica, Germany). Possible contaminations were also checked using the above described protocol.

### Statistical analysis

Growth curves were obtained with the percentages of covered surface for each medium and each strain, obtained from the analysis of all the photographs. A linear mixed-effects model was used to make comparisons between the different observed growth curves. If *y*_*ij *_denotes the *j*-th observation of the *i *strain then we assumes that:

(1) *y*_*ij *_= *β*_1_*t*_*ij *_+ *b*_1_*t*_*ij *_+ *ε*_*ij *_

where *t*_*ij *_is *j*-th observation time of the *i*-th strain. The parameter *β*_1 _corresponds with the mean slope meanwhile the random variable *b*_1 _(with normal distribution) corresponds with the random slope. It is assumed that *b*_1 _has a normal distribution with null mean and standard deviation *σ*_*b*1_. The error term in the model, *ε*_*ij *_is assumed independent and normally distributed (with variance *σ*_2_) for different times and different species. Additional details of the model used can be found in [9]. The statistical analysis was performed by using the NLME software package [10] and, particularly, for linear mixed-effect models, it has been used the package [11].

## Results

### Sliding motility

**Figure 1:**
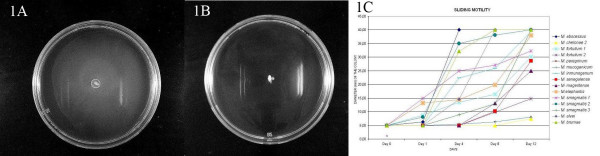
**Sliding motility of some strains.** 1A: *M. abscessus *(DSM 44196T). 1B: *M. septicum *(ATCC 700731T). 1C: Diameter of the colony in the motility experiments. *M. chelonae 1*: ATCC 19235;*M. chelonae 2*: ATCC 35752T; *M. fortuitum 1*: ATCC 6841T; *M. fortuitum 2*: ATCC 13756; *M. smegmatis 1*: ATCC 607; *M. smegmatis 2*: ATCC 19420T; *M. smegmatis 3*: ATCC 14468.

**Figure 2:**
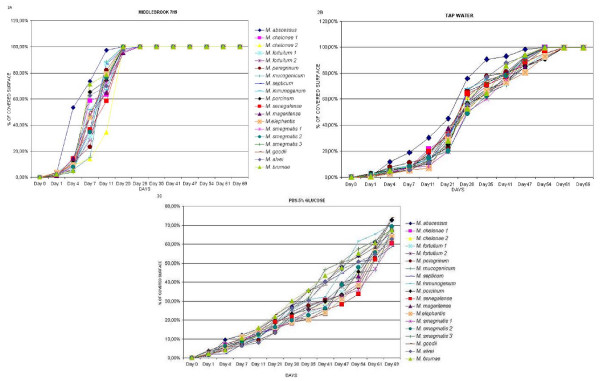
**Growth on non-pigmented rapidly growing mycobacteria strains on the different media:** 2A: Middlebrook 7H9. 2B: Sterile tap water. 2C: PBS-Glucose 5%. *M. chelonae 1*: ATCC 19235; *M. chelonae 2*: ATCC 35752T; *M. fortuitum 1*: ATCC 6841T; *M. fortuitum 2*: ATCC 13756; *M. smegmatis 1*: ATCC 607; *M. smegmatis 2*: ATCC 19420T; *M. smegmatis 3*: ATCC 14468.

All strains with the exception of *M. goodii*, *M. chelonae *ATCC 19235, *M. porcinum *and *M. septicum *showed sliding motility on Middlebrook 7H9-0.3% agar plates at the 7^th ^day (Figure 1). There were differences in the speed of sliding motility between strains, *M. chelonae *ATCC 35752T was found to be the slowest and *M. abscessus *the fastest (Figure 2D).

### Biofilm development

The reproducibility of the test was confirmed using the *M. smegmatis *strains and testing them three times each. All three strains produced biofilm that covered almost identical percentages of the surface and had identical growth patterns. No difference in biofilm growth was found between this first reproducibility experiment and the following experiment where *M. smegmatis *strains were tested together with all NPRGM tested. Data reported here for *M. smegmatis *are those obtained in the experiment where all species were tested. All strains tested developed biofilm with all media.

**Figure 3:**
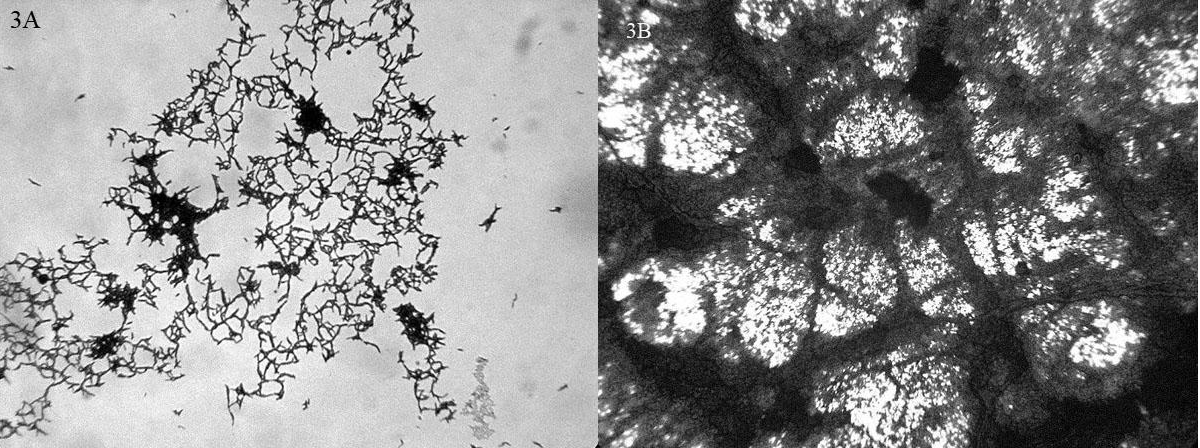
Morphology of biofilm development of *M. fortuitum *ATCC 6841T the day 4 (3A) and the day 24 (3B) in Middlebrook 7H9.

The strains showed a sigmoid growth curve during the development of the biofilm (Figure 2A) with Middlebrook 7H9. *M. abscessus *was the fastest species, although no significant differences between them could be detected. The strains grew in Middlebrook 7H9 showing initially a characteristic lace-like pattern, where bacteria were detected forming delicate nets of organisms (figure 3A). This pattern disappears when bacterial growth covers considerable parts of the surface (Figure 3B). All the strains covered 100% of the surface the 28^th ^day of incubation.

When sterile tap water was used as culture media, all the strains showed also a sigmoid growth curve (Figure 2B), however growth was slower and 100% of the surface was not covered until the day 63. Wells containing PBS-5% glucose had the worst results for biofilm development because none of the tested strains grew enough to cover all the surface even by day 69 (Figure 2C).

Table 1 displays the models fitted. The first column (Middlebrook 7H9, PBS-5% Glucose and Motility) indicates the data set meanwhile the model fitted is given in Equation 1. Basically, a random line passing through the origin plus a random error. The random slope has a normal distribution with mean *β*_*time *_and standard deviation *σ*_*time*_.

**Table 1:** Summary of the fitted models.

	*σ*_*time*_	*σ*_*residual*_	*β*_*time*_	*β*_*type*_
Middlebrook 7H9	4.63e-05	16.00	4.31	
Tap water	8.64 e-06	7.79	1.77	9.25
PBS-5% Glucose	0.08	3.89	0.88	
Motility	1.30	5.87	2.24	

The estimators for the different data sets appear in table 1 in the column headed *β*_*time *_and *σ*_*time*_. Note that the standard deviation of this random slope for Middlebrook 7H9 is almost null and, in fact, we have a fixed slope estimates as 4.31, i.e. for each additional day we observe a mean increment of 4.31% of covered surface for Middlebrook 7H9.

Analogous interpretations can be given for the estimates of column *β*_*time *_corresponding to PBS-5% Glucose and Motility (third and fourth rows). However, the mean increment is estimate as 0.88 for PBS-5% Glucose and the corresponding standard deviation is estimated as 0.08. For the motility the estimate is 2.25 but we have a large standard deviation estimated, 1.30. Additionally, the estimates of the standard deviation of the random error are shown in column headed *σ*_*residual*_. Note the large standard deviation estimated for Middlebrook 7H9 and Motility.

It can be observed that the row corresponding to tap water has an additional column headed *ψ*_*type*_. From the observation of the original data and by fitting a different linear model it was suggested that the behaviour of *M. abscessus *in this medium could be different from the other ones. We have introduced in the model an additional explanatory variable. The model now considered is:

(2) *y*_*ij *_= *β*_*time*_*t*_*ij *_+ *ψ*_*type*_*type *+ *b*_1_*t*_*ij *_+ *ε*_*ij *_

where *type *= 1 corresponds with *M. abscessus *and zero otherwise and *ψ*_*type *_is a constant. This model considers that the random slope can have a different mean for *M. abscessus *and a common but different mean for the other species. The mean of *M. abscessus *would be *β*_*time*_+ *ψ*_*type *_and for the other species would be *β*_*time *_respectively. This random slope will have a common standard deviation *σ*_*time*_. We have compared, for the tap water the two models, given in equations 1 and 2. The p-value observed is lesser than 0.0001, i.e. the mean of the random slope for *M. abscessus *is significatively different from the other species in tap water. In particular, the mean increment per unit time would be 1.78+9.25 = 11.02 for *M. abscessus *and 1.77 for the other ones. Note that the standard deviation is almost null for the tap water data, i.e. we can consider a constant slope estimated with the values just commented. Finally a large standard deviation of the random error is estimated, 7.79.

**Figure 4:**
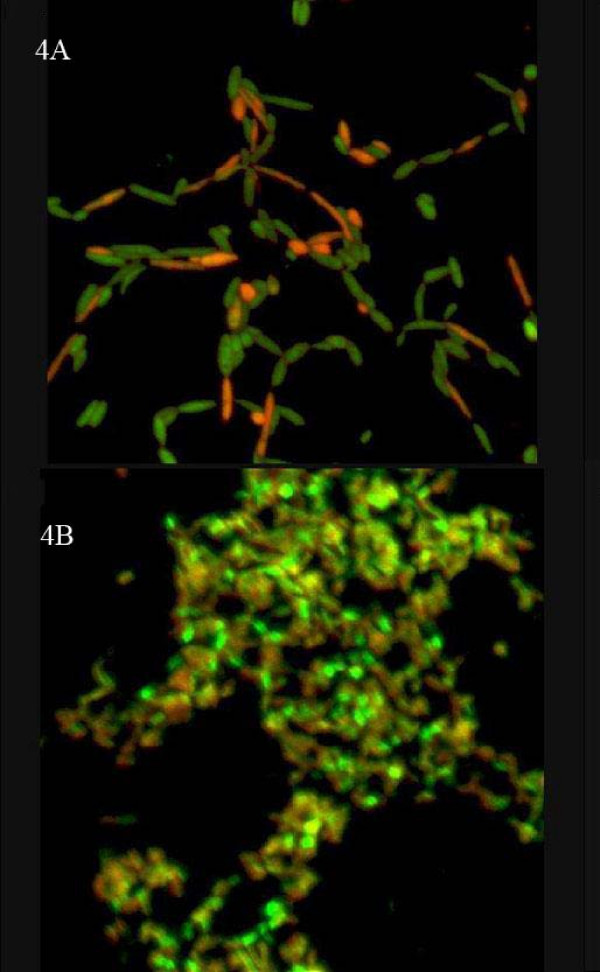
**CLSM of mycobacterial biofilm.** 4A: *M. fortuitum *(day 7, 100×), 4B: *M. septicum *(day 20 40×).

All the strains analysed by CLSM developed biofilm. The photographed structures showed identical growth patterns than those observed with fuchsine stain (Figure 4). Live/dead^© ^stain allow us to evaluate the presence of live and dead bacteria in the biofilm, with higher percentages of live bacteria in early states of biofilm development (75–80% of live bacteria the day 7) than in the mature biofilm (approximately 50% of live bacteria the day 20). At day 24, biofilm had a thickness of around 20 μm in all tested species.

## Discussion

The relationship between biofilms and mycobacteria has been known for many decades, both in environmental and in medical settings. Detection of mycobacteria in biofilm samples from different water systems have been reported [1,4,5,12-15]. However, in these reports identification of the species was not achieved in all cases. Rapidly growing species, such as *M. fortuitum *and *M. chelonae*, have been described as part of these polymicrobial biofilms, where slowly growing mycobacteria have also been isolated. Recent taxonomic changes among this group of organisms, however, make the former identifications less valuable, because many of the recently characterized species were identified as *M. chelonae *or *M. fortuitum *according to old identification schemes [1].

The clinical significance of the isolates of NPRGM is unclear, with a high percentage of NPRGM being identified as "contaminants" or "colonizers", especially related with the origin of the sample [16]. Infections due to NPRGM include a broad range of diseases, the majority of them being caused by 3 of these species (*M. fortuitum*, *M. chelonae *and *M. abscessus*), with all other species rarely causing human infection [1,17-19], despite their relatively common isolation from clinical samples [1,3,16,20,21]. These pathogenic infections are commonly biofilm-related ones, and include a large variety of surgical complications which ranged from relatively mild infections, such as surgical wound infections [1,22], to extremely severe ones, such as prosthetic valve endocarditis [23,24]. In our hospital, biofilm-related infections represent half of the infections caused by NPRGM since 1980 to 2003 [3] and this percentage increased in a more recent multicenter study performed in our area [23]. Few reports evaluate *in vitro *biofilm development in NPRGM [8,25-36], although data concerning the detection of such mycobacteria in clinical samples has been published many years ago [37-40]. Recently, our laboratory described the ability of several strains of NPRGM to attach to polypropylene sutures, a first step in the development of biofilm [41]. Because biofilm development is a common pathogenic factor for many bacteria, and because the differences in clinical significance of the different species of NPRGM, we studied NPRGM of clinical interest to see if there were any differences in biofilm development. We found that all species tested can develop a biofilm, so this characteristic is not species-specific. However, we have found a faster growth of *M. abscessus *in one of the tested media. Because this species is probably the most pathogenic one [1,2,16,42], this difference can represent a property that makes this species more pathogenic than others, despite all of tested NPRGM are able to develop a biofilm. *M. abscessus *has been described as the cause of many biofilm-related diseases, and recent reports showed that the ability to develop biofilm seems to be related with other pathogenic mechanisms, such as colony phenotype or virulence in experimental models [29].

In this study biofilm development of representative NPRGM species was analyzed using several techniques. One of these techniques has been developed in our laboratory based in previously described systems [33]. It allows us to follow the dynamics of biofilm development, including an analysis of the morphology of the growing biofilm. For all NPRGM, a sigmoid growth curve was seen in two of the tested media, and a similar curve has been previously described for *M. fortuitum *using a different technique [27].

Another finding deals with the changes in biofilm development when using different media. A previous study [28] showed that low nutrient conditions decrease biofilm development for *M. fortuitum *and *M. chelonae*. In our study, we detected that such modifications in biofilm development could be detected among all the tested species. Tap water was found to be a better culture media than PBS-glucose for biofilm development. This difference can be due to the fact that tap water has many chemical molecules in minimal quantities that could be of great importance as nutrients for many organisms, including mycobacteria [4,43]. Another important issue could be the temperature. We select temperature for attachment based on a previous study performed by us [41], and we then select room temperature as the incubation temperature because it is the temperature where biofilms caused by these mycobacteria grows in natural environments. However, further experiments must be done to assess if temperature changes can affect the biofilm development. This factor can also influence the motility, because some species have also different optimal temperatures for growth. We selected 37°C for the motility experiment based on previously published studies [8].

Of interest, we have detected important differences in sliding motility among the studied strains, despite the fact that all of them can develop biofilm. Because these two properties have been linked previously [8,29,31,32], and related with the presence of glycopeptidolipids in mycobacterial cell wall [8,31], we speculate that there could be differences in the lipid content of the cell wall of the different species that can affect the results of the experiment. In our study, some representative strains did not show sliding motility, so it seems that this property is not always related with biofilm development, as has been recently described for *Mycobacterium avium *[44].

## Conclusion

All the tested species of NPRGM are able to form biofilm in vitro, so this property is not species-specific in this group of mycobacteria. The chemical composition of the media influences the time necessary for the biofilm development of all tested species. The protocol described here allows is easy to perform and allows following the biofilm development and is useful for further studies in this field.

## Authors' contributions

JE coordinate all the study. Conceived and participate in the design the study, and participates in the analysis of the results. NZM perform the experimental study and collaborates in the analysis of the results. TJK participates in the analysis of the results and helped to draft the manuscript. GA perform the statistical analysis. RFR participates in the design of the study. IG participates in the design of the study and in the statistical analysis. All the authors participate in the redaction of the manuscript, have read it and approved it.
